# Construction Notes

**Published:** 1894-10-20

**Authors:** 


### CONSTRUCTION NOTES.

**Cottage Hospital at Berriedale.**

The Duke and Duchess of Portland have opened a cottage hospital for the benefit of their tenants at Langwell. This consists of a six-roomed cottage, situated close to the sea. The hospital is divided into two floors, two wards and a nurses' room being upstairs, and a day-room, kitchen, and laundry on the ground floor. The nurses' room is situated between the two wards. There is a well-stocked linen cupboard and all nursing requisites.

**Fleetwood Infirmary.**

This new hospital now in course of erection in Pharos Street will eventually be replete with every convenience for coping with the accidents which are of frequent occurrence in the town of Fleetwood and the large industrial concerns in the immediate vicinity. The building has a north aspect. A large garden 110 feet will form a frontage. The hospital front is built of red stone and red pressed brick, and there will be a small balcony over the main entrance. Immediately on the right to the main entrance in Pharos Street is the committee room, 17 by 12 feet; opposite to this is the matron's room. On the same ground floor there is also a bath-room, bed-room, and two large wards, one on each side of the passage. These wards are 12 feet high, and are provided with four beds each, though there is room for five beds if required. Proceeding further inwards we found the surgery and operating-room, with a good kitchen, scullery, pantry, and washhouse. On the ground floor will be found two wards, 10 by 13 feet, for two beds each, and there are also three bed-rooms and bath-room on this floor. Messrs. Drummond and Sons have done their work in an efficient manner, and the outcome is a neat and substantial building. The cost of same, including furnishing, is estimated at about £2,500, close upon £1,000 being yet required.

**Mr. Quarrier's Consumptive Home.**

The memorial stone of this new consumptive home has been laid at the Bridge of Weir. The site, which adjoins that on which the orphan homes are erected, is on ground sloping towards the south. There will be eight or ten buildings (according as funds permit) consisting of hospital homes with a doctor's house, a store, a separate hospital, a washing-house and laundry, and rooms for the maids. It is expected that the buildings will be ready for occupation within a year. The erection of the first building, which is intended for accommodating women and children, has been already begun. The style which the architect, Mr. R. A. Bryden, has adopted is plain gothic. There will be a projecting roof on the hospital, the walls will be hollow, and the windows will have inner sashes so as to keep the air at a uniform temperature. The floors will be of pitch pine, no skirtings or projecting woodwork will be anywhere allowed. The building will be partly two stories and partly three in height, while the basement will contain the heating and ventilating apparatus. The ventilation will be on the propulsion system from a fan in the basement floor. On the ground floor will be a large recreation-room, a corridor for exercising patients, and nine wards and single apartments for patients' use. Also a matron's parlour, attendants' room, &c. The first floor contains a dining-room, a quiet room, and exercise room, and nine wards and single apartments, with matron's bed-room, &c. The upper floor will contain the kitchen, which is in the centre, the scullery, the store room, with two wards for convalescents and two bed-rooms for servants. All these rooms, with the exception of four, face south, and ten apartments are for single patients only. The rooms provide 1,200 cubic feet of air space per patient. The best-known consumptive homes have been visited in England by Mr. Quarrier and his architect, and all the fittings and materials known to science as available for the equipment of such buildings will be adopted in this, the first consumptive hospital in Scotland. The estimated cost of the building is £7,000, and the contractors are Messrs. George Barlas and Co. Heating and ventilating, James Carnack and Sons.

